# Paediatric caecal volvulus, a rare presentation of african degenerative leiomyopathy – a case report

**DOI:** 10.1007/s00384-026-05093-y

**Published:** 2026-01-26

**Authors:** Francesca Palmisani, Emanuele Trovalusci, Sphamandla Zulu, Seo-Hwa Chung, Leila Hartford, Giulia Brisighelli

**Affiliations:** 1 Department of Paediatric Surgery, Medical University of Vienna, Vienna, Austria; 2 Johannesburg Paediatric Colorectal Clinic, Chris Hani Baragwanath Academic Hospital, Department of Paediatric Surgery, University of the Witwatersrand, Johannesburg, South Africa; 3 Department of Women’s and Children’s Health, University of Padua, Padua, Italy

**Keywords:** African degenerative leiomyopathy, Paediatric intestinal pseudo-obstruction, Volvulus, Hollow visceral myopathy, Case report

## Abstract

**Background:**

Caecal volvulus is a rare condition with an unknown prevalence, particularly in paediatric patients. Predisposing factors include fixation anomalies of the colon (with or without malrotation) and significant intestinal distension due to conditions such as chronic constipation, post-operative ileus, Hirschsprung disease (HD), or paediatric intestinal pseudo-obstruction (PIPO). African degenerative leiomyopathy (ADL) is a regional variant of visceral myopathy characterised by a fibrotic “tiger-striped” degeneration of the muscular layers of the colon, which causes PIPO. It is endemic to Sub-Saharan Africa and is associated with poor outcomes. To our knowledge, this is the first case in which a caecal volvulus led to the diagnosis of ADL.

**Case presentation:**

An 11-year-old female, previously healthy, presented with a 2-day history of abdominal distension, bilious vomiting, and constipation. Abdominal radiography and a computed tomography (CT) scan were suggestive of a colonic volvulus. Endoscopic reduction was unsuccessful, and exploratory laparotomy revealed a caecal volvulus. A limited right hemicolectomy and end ileostomy were performed. Histology revealed the typical myopathic changes with “tiger-striped” fibrosis and atrophy. Postoperatively, she experienced recurrent episodes of bowel pseudo-obstruction. A full-thickness rectal biopsy confirmed the presence of ganglion cells, excluding HD and further supporting the diagnosis of ADL. The patient, unfortunately, died 6 months later due to abdominal compartment syndrome.

**Conclusions:**

ADL is a rare and often fatal condition associated with intermittent bowel obstruction and systemic complications, including cardiac and urologic abnormalities. In paediatric patients, caecal volvulus should prompt consideration of PIPO as an underlying diagnosis.

**Supplementary Information:**

The online version contains supplementary material available at 10.1007/s00384-026-05093-y.

## Introduction

In adult populations, colonic volvulus accounts for 10–15% of all large bowel obstructions, with a slightly higher incidence in Africa, India, and the Middle East (so-called volvulus belt). Any segment of the colon may be affected, but it is mostly reported to involve the sigmoid colon (60–75%) and the caecum (25–40%), with the latter presenting predominantly in younger females [1, 2].

In paediatric patients, the incidence of caecal volvulus remains unknown, due to the scarcity of reported cases. In children, caecal volvulus is likely associated with abnormal mobility of the caecum, which may be due to a congenital failure of parietal fixation during embryologic development (malrotation) or to chronic dilatation of the bowel with stretching of the mesentery, such as in cases of chronic constipation or chronic ileus [2, 3].

Chronic intestinal pseudo-obstruction (CIPO) is a rare disorder characterised by recurrent episodes of ileus in the absence of an identifiable mechanical or anatomical cause [4]. In 2018, an European Society for Paediatric Gastroenterology, Hepatology and Nutrition (ESPGHAN)-led expert group has proposed the term paediatric intestinal pseudo-obstruction (PIPO) to address the large spectrum of gut motility disorders that affect specifically the paediatric population [5]. The aetiology of PIPO can be primary (sporadic or familial), secondary, or idiopathic [6]. In children, neuropathic forms are more common (approximately 70%) compared with the adult population, while myopathic forms are seen in approximately 30% of cases [5]. The reason for the distinction from the adult entity is that these conditions are often congenital and may involve other organs, including the bladder and heart. Furthermore, the poor prognosis of the disease, which has a reported mortality rate of up to 30% in childhood, calls for a multidisciplinary effort to better understand its pathophysiology and improve timely diagnosis and management [5].

With this in mind, we present a case of caecal volvulus in an 11-year-old girl that led to the diagnosis of PIPO.

This study adhered to the case report (CARE) guidelines.

## Case description

An 11-year-old female was referred to our centre from a district hospital with a 2-day history of abdominal distension, bilious vomiting, and constipation. Her caregiver reported normal growth and no history of chronic constipation or diarrhoea. She had passed meconium within the first 24 h of life, and there was no family history of gastrointestinal disease.

On examination, the patient was haemodynamically stable. Her abdomen was distended but soft, and bilious content was draining via a nasogastric tube.

**Fig. 1:**
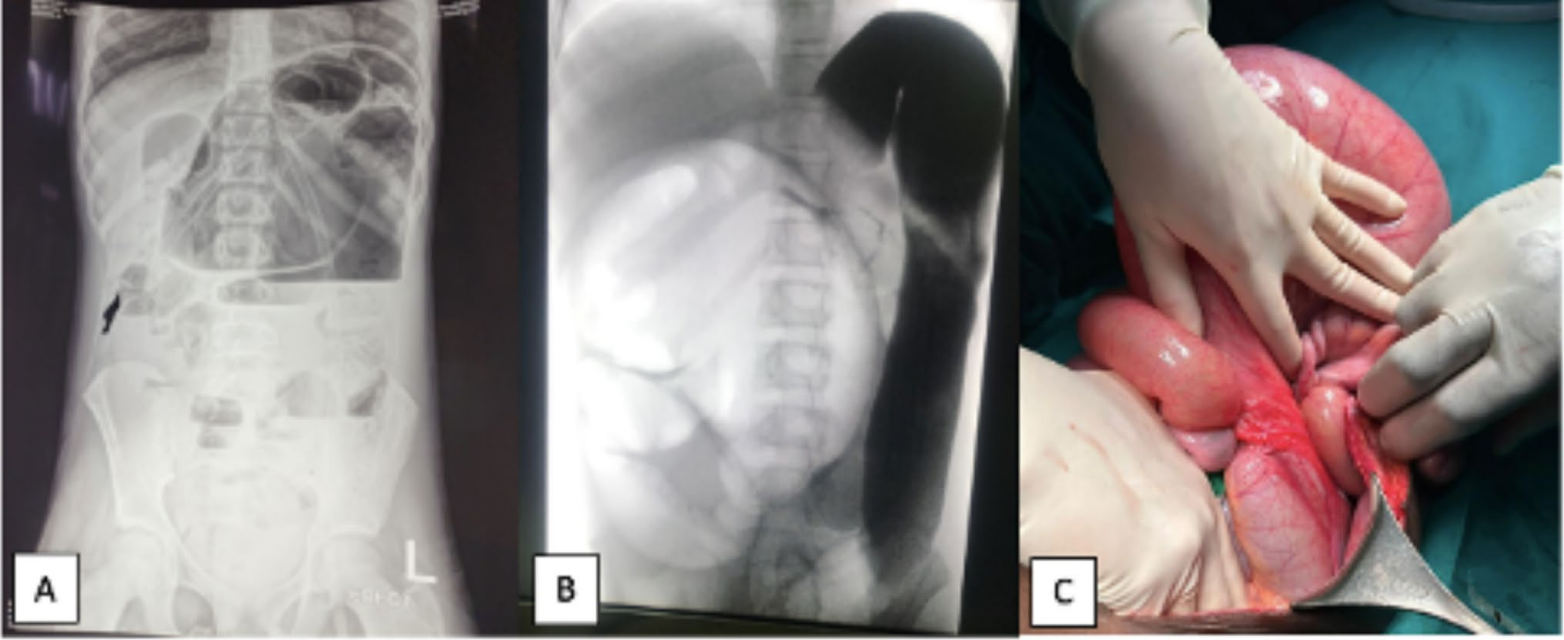
**A** Abdominal X-ray at presentation: note the dilated loops of the bowel in the left upper quadrant. **B** Contrast enema: contrastunable to pass the splenic flexure and kidney bean sign in the right lower quadrant. **C** Intraoperative findings of caecal volvulus

Abdominal radiography revealed dilated loops of bowel (Fig. 1A), particularly in the left upper quadrant, with a paucity of distal air. A computed tomography (CT) scan of the abdomen was performed, showing features suggestive of a sigmoid volvulus.

An urgent colonoscopy was performed to attempt an endoscopic detorsion. The colonoscope was advanced to the splenic flexure, without evidence of torsion or ischaemia. As the patient’s abdominal distension improved during the procedure, a conservative approach was adopted. However, the following day she deteriorated, with worsening abdominal distension. An urgent contrast enema was performed (Fig. 1B) revealing failure of the contrast to pass the sigmoid flexure, and a “kidney bean sign” in the right lower quadrant, raising suspicion for caecal volvulus. This configuration results from a closed-loop obstruction and twisting of the colon, causing the opposed walls of the distended bowel to form the central cleft. In sigmoid volvulus, this stripe typically points towards the left lower quadrant, while in a caecal volvulus, the orientation is towards the right lower quadrant. The American College of Surgeons describes this sign as a key radiographic finding that should prompt further evaluation for volvulus [1].

An emergency laparotomy confirmed the diagnosis (Fig. 1C). The volvulus was detorsed, and a limited right hemicolectomy with end ileostomy was performed. A full-thickness rectal biopsy was also taken to exclude HD. Her recovery was complicated by a prolonged ileus, which required the insertion of a central line for parenteral nutrition. A nasogastric tube (NGT) was left in place for 6 days due to recurrent postoperative bilious vomiting. She slowly recovered and, after the return of bowel function on day 7, enteral feeds could be progressively introduced and were subsequently tolerated. She was discharged on day 12 postoperatively. Five days later, she was readmitted with abdominal distension and absent stoma output. An abdominal X-ray and a contrast swallow with follow-through showed signs of adhesive bowel obstruction, for which she required a second laparotomy and adhesiolysis; however, no obvious intra-abdominal mechanical obstruction was identified. The ileostomy was left in place. Once again, the postoperative course was complicated by a prolonged ileus, and enteral feeding was only re-established on day 11 postoperatively. Anticipating this possible complication, a central line had been inserted at the time of surgery, and parenteral nutrition was started immediately postoperatively. No admission to the intensive care unit was deemed necessary.

**Fig. 2:**
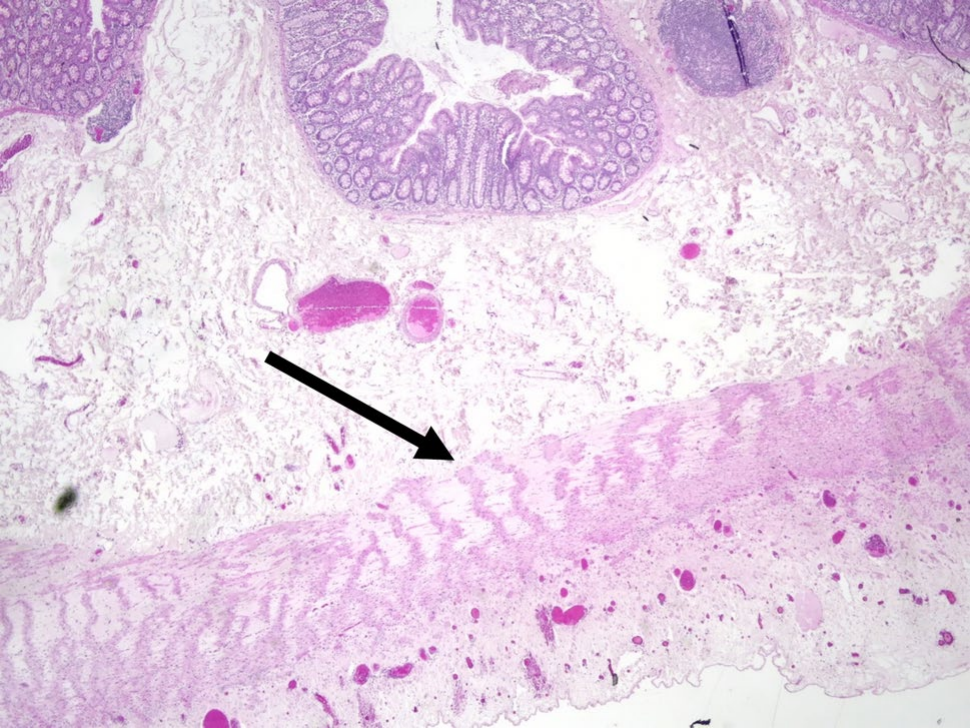
Haematoxylin and eosin-stained section of the large bowel showing fibrosis of muscularis propria in a “tiger-stripe” pattern (arrow), with marked atrophy and patchy vacuolar degeneration of the outer longitudinal layer also visible

Histology (haematoxylin and eosin stain) excluded HD, confirming the presence of ganglion cells (Fig. 2). However, vacuolisation of smooth muscle cells with fibrosis of the muscularis propria with a tigroid appearance, together with marked atrophy of the outer longitudinal layers, suggested a myopathy consistent with African degenerative leiomyopathy (ADL) (further supported by Masson’s trichrome stain). These findings were present both in the rectal biopsy and in the resected colonic specimen, while the appendix and the terminal ileum were normal. Further investigations, including echocardiography and urinary tract ultrasound, revealed no associated anomalies. Genetic testing was recommended but not conducted due to financial limitations. Due to the known poor functional outcome of ADL, no ileostomy reversal was planned. The patient was started on a conservative management plan based on laxatives (senna) and stool softeners, as well as dietary recommendations and nutritional supplements. These therapeutic interventions were reported to be well tolerated by the patient and the family. Daily stoma care was also not described as an issue, and could be routinely carried out by the patient herself.

However, over the next several months, despite having an end ileostomy, the patient was admitted multiple times with pseudo-obstructive symptoms that responded to conservative management. Six months after initial presentation, and despite ongoing outpatient follow-up, she acutely developed abdominal compartment syndrome secondary to severe bowel distension, resulting in multi-organ failure and metabolic acidosis. She died shortly thereafter in casualty from cardiac arrest. No post-mortem examination was performed in accordance with family preference.

## Discussion

Hollow visceral myopathy (HVM) is a term first used in 1985 to describe a rare cause of chronic intestinal pseudo-obstruction in black South Africans. Also known as ADL, it is a region-specific condition predominantly affecting young Africans in Southern, Central, and East Africa [7]. It is characterized by a myogenic degeneration of intestinal smooth muscle that results in progressive functional obstruction, primarily affecting the colon and advancing proximally. Histologically, the absence of vacuolated mitochondria and the pattern of cytoplasmic translucency observed on electron microscopy distinguish ADL from other forms of PIPO, as well as from other connective tissue diseases of childhood [8]. Another distinguishing feature is the later onset, with clinical symptoms typically appearing at a median age of 9.5 years, rather than during the first years of life [9].

As with all PIPOs, it is considered an orphan disease, with an unknown incidence [10]. Literature regarding this form indeed consists of only a few case series and case reports, with the broadest consisting of 39 patients from the Western Cape in South Africa [8].

Although ADL has traditionally been considered an acquired condition, possibly due to environmental exposures (e.g. herbal enemas containing compounds such as dichromates), recent evidence suggests genetic associations and familial cases [11, 12]. In one study, 60% of patients with ADL had a variant in exon 5 of the ACTG2 gene—a gene involved in the actin–myosin apparatus, frequently associated with other forms of HVM–PIPO. However, it remains unclear whether these genetic variants are clinically relevant [11].

The resource-limited context of low- and middle-income countries adds to the diagnostic challenge of orphan diseases: the virtual absence of genetic testing, forcing reliance primarily on the clinical picture and therefore on the experience of the attending physicians.

ADL typically presents with recurrent abdominal pain, distension, and vomiting. The condition causes malabsorption due to impaired intestinal function and bacterial overgrowth, leading to malnutrition and a progressive decline in health. Involvement of the urinary bladder and myocardium is common but tends to occur later than in other PIPOs [8]. Diagnosis is suspected based on the typical clinical presentation and radiologic findings of pseudo-obstruction, and is confirmed by characteristic findings on intestinal biopsies [4, 8]. Histopathology revealing marked atrophy and the characteristic “tiger-striped” appearance, which represents a fibrotic replacement of the intestinal smooth muscle, is essential for confirming ADL and distinguishing it from other forms of HVMs or neuronal forms of PIPO.

Colonic volvulus has already been described as a possible complication in patients with known intestinal motility disorders [5]. However, existing literature has primarily focused on the diagnostic challenge of distinguishing mechanical obstruction due to volvulus from the functional pseudo-obstruction episodes typically seen in these patients, which do not require surgical intervention [4, 13].

To our knowledge, this is the first reported case in which a colonic volvulus served as the initial presentation leading to the diagnosis of an underlying intestinal motility disorder.

The stasis of intestinal contents and chronic dilatation of the colon may be precipitating factors in colonic volvulus, and further investigations are warranted to identify the underlying cause. Following the recommendations of the ESPGHAN study group, once HD and other causes of mechanical obstruction are excluded, PIPO should be considered a possible diagnosis, and the patient should undergo a thorough evaluation to exclude any other hollow visceral involvement [5]. Unfortunately, genetic testing, which could have added relevant information to this case report, was not performed due to resource constraints, limiting our possibility to contribute further in unraveling the pathogenesis of ADL.

Due to the poor prognosis, early multidisciplinary input, nutritional support, and, where possible, early palliative care involvement are crucial.

Management strategies for ADL remain controversial, as no regimen has so far been proven to be of lasting value. Medical treatment is based on irrigations, laxatives, and prokinetic agents, as well as a low-residue diet, which, however, is effective only in the initial phases of the disease, as over time the muscular degeneration and fibrotic replacement limit the overall bowel response [8]. 

For this reason, the adoption of surgical strategies such as Malone antegrade continence enema stomas (MACE) or button cecostomies has been described to target gaseous distension as well as chronic constipation [14]. According to recent literature, this may result in symptomatic relief and at least temporary improvement in quality of life [14]. 

ADL, however, in contrast to other forms of myopathy, is currently described as incurable, and all management strategies are considered palliative. Long-term parenteral nutrition, which might address the inherent malnutrition that accompanies ADL, is not described as a viable option, possibly due to the resource constraints of the regions in which it is endemic.

## Conclusion

ADL is a rare and often fatal regional variant of PIPO with systemic complications, predominantly affecting young individuals in Southern, Central, and Eastern Africa.

In the absence of other known underlying aetiologies such as HD, caecal volvulus should prompt consideration of PIPO as a potential underlying diagnosis.

This report aims to expand the currently limited body of evidence on this orphan disease, highlighting its diagnostic and therapeutic challenges, especially in low- and middle-income countries.

## Supplementary Information

Below is the link to the electronic supplementary material.

ESM1 (PDF 630 KB)

## Data Availability

The datasets analysed during the current study are available from the corresponding author upon reasonable request.

## Abbreviations

HD: Hirschsprung disease
PIPO: Paediatric intestinal pseudo-obstruction
ADL: African degenerative leiomyopathy
CT: Computed tomography
CIPO: Chronic intestinal pseudo-obstruction
HVM: Hollow visceral myopathy
