# Supplementary material for: Paediatric caecal volvulus, a rare presentation of african degenerative leiomyopathy – a case report
Source: Int J Colorectal Dis. 2026 Jan 26;41(1):46. doi: 10.1007/s00384-026-05093-y (PMC12835031; doi:10.1007/s00384-026-05093-y)
Supplement: Supplementary file 1 — (PDF 630 KB) [file 384_2026_5093_MOESM1_ESM.pdf]

CARE
case report guidelines

CARE Checklist of information to include when writing a case report

(cc) BY-NC-ND

Topic	Item	Checklist item description	Reported on Line
**Title**	1	The diagnosis or intervention of primary focus followed by the words "case report"	3
**Key Words**	2	2 to 5 key words that identify diagnoses or interventions in this case report, including "case report"	59-60
**Abstract (no references)**	3a	Introduction: What is unique about this case and what does it add to the scientific literature?	43-44
	3b	Main symptoms and/or important clinical findings	46-48
	3c	The main diagnoses, therapeutic interventions, and outcomes	48-54
	3d	Conclusion—What is the main "take-away" lesson(s) from this case?	57-58
**Introduction**	4	One or two paragraphs summarizing why this case is unique (**may include references**)	79-97
**Patient Information**	5a	De-identified patient specific information.	103-106
	5b	Primary concerns and symptoms of the patient.	103-104
	5c	Medical, family, and psycho-social history including relevant genetic information	105-106
	5d	Relevant past interventions with outcomes	104-105
**Clinical Findings**	6	Describe significant physical examination (PE) and important clinical findings.	107-108
**Timeline**	7	Historical and current information from this episode of care organized as a timeline	103-157
**Diagnostic Assessment**	8a	Diagnostic testing (such as PE, laboratory testing, imaging, surveys).	109-111; 113-122
	8b	Diagnostic challenges (such as access to testing, financial, or cultural)	145
	8c	Diagnosis (including other diagnoses considered)	124; 139-142
	8d	Prognosis (such as staging in oncology) where applicable	146
**Therapeutic Intervention**	9a	Types of therapeutic intervention (such as pharmacologic, surgical, preventive, self-care)	124-150
	9b	Administration of therapeutic intervention (such as dosage, strength, duration)	124-150
	9c	Changes in therapeutic intervention (with rationale)	NA
**Follow-up and Outcomes**	10a	Clinician and patient-assessed outcomes (if available)	NA
	10b	Important follow-up diagnostic and other test results	152-157
	10c	Intervention adherence and tolerability (How was this assessed?)	147-150
	10d	Adverse and unanticipated events	152-157
**Discussion**	11a	A scientific discussion of the strengths AND limitations associated with this case report	198-206
	11b	Discussion of the relevant medical literature **with references**.	162-221
	11c	The scientific rationale for any conclusions (including assessment of possible causes)	225-228
	11d	The primary "take-away" lessons of this case report (without references) in a one paragraph conclusion	227-230
**Patient Perspective**	12	The patient should share their perspective in one to two paragraphs on the treatment(s) they received	NA
**Informed Consent**	13	Did the patient give informed consent? Please provide if requested	Yes ☑ No ☐
